# 100 pT/cm single-point MEMS magnetic gradiometer from a commercial accelerometer

**DOI:** 10.1038/s41378-020-0173-z

**Published:** 2020-08-10

**Authors:** Josh Javor, Alexander Stange, Corey Pollock, Nicholas Fuhr, David J. Bishop

**Affiliations:** 1 Department of Mechanical Engineering, Boston University, Boston, MA 02215 USA; 2 Division of Materials Science and Engineering,, Boston University, Boston, MA 02215 USA; 3 Department of Electrical and Computer Engineering, Boston University, Boston, MA 02215 USA; 4 Department of Physics, Boston University, Boston, MA 02215 USA; 5 Department of Biomedical Engineering, Boston University, Boston, MA 02215 USA

**Keywords:** Sensors, Nanometrology

## Abstract

Magnetic sensing is present in our everyday interactions with consumer electronics and demonstrates the potential for the measurement of extremely weak biomagnetic fields, such as those of the heart and brain. In this work, we leverage the many benefits of microelectromechanical system (MEMS) devices to fabricate a small, low-power, and inexpensive sensor whose resolution is in the range of biomagnetic fields. At present, biomagnetic fields are measured only by expensive mechanisms such as optical pumping and superconducting quantum interference devices (SQUIDs), suggesting a large opportunity for MEMS technology in this work. The prototype fabrication is achieved by assembling micro-objects, including a permanent micromagnet, onto a postrelease commercial MEMS accelerometer using a pick-and-place technique. With this system, we demonstrate a room-temperature MEMS magnetic gradiometer. In air, the sensor’s response is linear, with a resolution of 1.1 nT cm^−1^, spans over 3 decades of dynamic range to 4.6 µT cm^−1^, and is capable of off-resonance measurements at low frequencies. In a 1 mTorr vacuum with 20 dB magnetic shielding, the sensor achieves a 100 pT cm^−1^ resolution at resonance. This resolution represents a 30-fold improvement compared with that of MEMS magnetometer technology and a 1000-fold improvement compared with that of MEMS gradiometer technology. The sensor is capable of a small spatial resolution with a magnetic sensing element of 0.25 mm along its sensitive axis, a >4-fold improvement compared with that of MEMS gradiometer technology. The calculated noise floor of this platform is 110 fT cm^−1^ Hz^−1/2^, and thus, these devices hold promise for both magnetocardiography (MCG) and magnetoencephalography (MEG) applications.

## Magnetic sensing: Small, low-cost magnetic sensor with wide field strength

A small, low-power, inexpensive magnetic sensor has been developed in a commercially available platform with a resolution that is within the range of biomagnetic fields. Magnetic sensing is applied in everyday interactions with consumer electronics (such as for navigation) and has the potential to measure extremely weak biomagnetic fields (such as those of the heart and brain). Hitherto, biomagnetic fields have been measured only using expensive mechanisms. However, a team headed by Josh Javor at Boston University, USA has succeeded in applying the benefits of micro-electromechanical systems to fabricate a low-cost magnetic sensor in a small, versatile platform that can be easily integrated into consumer technology. We believe that their new technology has the potential to revolutionize magnetic sensing and offer considerable advantages in such areas as navigation, communication, and biomagnetic field mapping.

## Introduction

Magnetic sensing spans many scientific applications, from consumer electronics to cutting-edge biomagnetic research. Smartphones utilize the Earth’s magnetic field for navigation. Automobiles leverage noncontact magnetic sensing to determine the position of components, such as in the crank shaft and braking systems. Hall effect sensors are commonly used in these applications due to their low cost and manufacturability but are often limited by Earth’s magnetic field, which has a strength of 50 µT (ref. ^1^). The highest-resolution magnetic sensors have been used to measure the biomagnetic fields of the brain and heart, which are at least a million times smaller than Earth’s field (approximately 100 pT for the heart and 200 fT for the brain)^1,2^. Biomagnetic sensing traditionally requires liquid-helium-cooled detection using superconducting quantum interference devices (SQUIDs) but can also be measured using optically pumped, atomic magnetometers^1–5^. Recent developments in other sensing technologies, such as magnetoresistive, fluxgate, and more, suggest this capability as well^6–9^. Most of these techniques, however, face the barrier of the Earth’s magnetic field because they measure a uniform field, i.e., a magnetic field that is unchanging in position. For biomagnetic measurements, this requires heavily shielded rooms (typically a 60 dB attenuation), averaging or triggering using EKG leads, and large costs (on the order of $10k per sensor; QuSpin). To fully realize the clinical capabilities of biomagnetic sensing, arrays including many sensors are needed for biomagnetic mapping, enhancing the impact of the cost and size^10^.

**Fig. 1:**
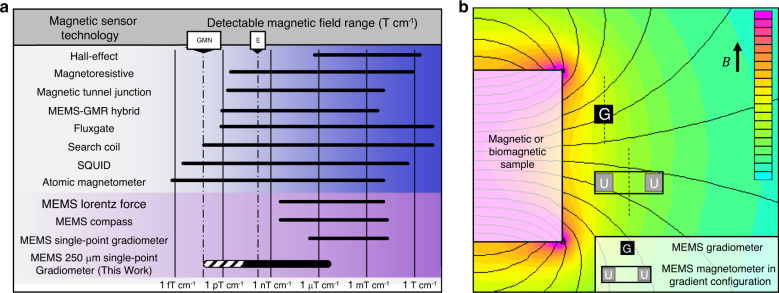
Existing Gradiometer Technology. **a** Resolution and range of various magnetic sensors 1, 2, 4–9, 11–19. E is Earth’s gradient field, and GMN is geomagnetic gradient noise. This work, an advance of MEMS technology (purple highlight), is shown at the bottom and compared to all magnetic sensors 3, 7, 8. The dark lines indicate the measurable range, while the dashed indicate the theory. This work represents the only intrinsically gradient field sensor. Uniform field sensor measurements are converted to gradient fields by assuming a separation of 1 cm. **b** Illustration showing how two uniform field sensors can measure a gradient field when signals are subtracted. A smaller separation between sensors or a single-point gradient measurement will allow the sensor to get closer to the source, where the fields are larger

The existing magnetic sensing technology is listed in Fig. 1a, where uniform field sensors are converted to gradient fields, to compare it to this work^1,2,4–9,11–19^. A gradient configuration is commonly, used where two magnetometers (sensitive to uniform magnetic fields) are arranged such that the two sensors are spaced some distance apart, as shown in Fig. 1b, and the signals are subtracted to find the spatial derivative of the magnetic field along a single axis^19^. All technologies in Fig. 1a are converted using a separation of 1 cm between sensors (assuming the sensors are small enough). Notably, a gradient field capable of being measured at a single point by a small sensor could be closer to a source, where fields are often larger as a cubic function of the distance (such as the case in Eq. 5, “Methods”). Additionally, a gradiometer is often useful to reduce geomagnetic noise, as the gradient of the Earth’s field noise is smaller (geomagnetic gradient noise is denoted by GMN in Fig. 1a).

Microelectromechanical system (MEMS) sensing technology offers many attractive advantages, such as a small size, low cost, and scalable fabrication. Several magnetic sensing mechanisms have been investigated, where the most common leverage is the Lorentz force or an MEMS compass design^11–19^. The best of the Lorentz force magnetometers (further details in Supplementary Table 1) achieves a resolution of 3.5 nT at 400 kHz (ref. ^11^). While this is impressive, many magnetic sensing applications are at lower frequencies (such as biomagnetism), requiring more development or techniques to modulate the field at the sensor’s resonance^6^. MEMS compassing technology usually involves the integration of a permanent magnet and then sensing of the torsional deflection on the magnet due to a magnetic field, similar to a compass^17–19^. The best of these integrated electron tunneling feedback controls can achieve a resolution of 300 pT Hz^−1/2^ at 1 Hz, but more development is necessary to increase fabrication repeatability to realize its potential^17^. Moreover, the footprint of this sensor is large (10 cm^2^), such that two of these sensors with minimal separation might measure a gradient field of 3 nT cm^−1^ (as reflected in Fig. 1a).

More recently, MEMS gradiometers have been investigated for the same benefits of traditional MEMS technology and the added benefit of reducing interference from the Earth’s field by measuring the gradient^20–24^. Here, both the gradient field resolution and spatial resolution (footprint) are important when measuring the positional change of a magnetic field. One technique is to fabricate two Lorentz force sensors close together on the same chip (separated by 3.9 mm), estimating a resolution of 100 µT cm^−1^ (ref. ^20^). Other works have developed so-called single-point gradiometers that do not require the subtraction of two uniform field sensors, circumventing the issues of asymmetry and drift^21–24^. These works typically integrate permanent magnets and have achieved resolutions in the high mT cm^−1^ range with large footprints (>1 mm^2^). Finally, an MEMS cantilever design with deflections measured by laser Doppler vibrometry was suggested to be capable of measuring gradient magnetic fields as low as 100 nT cm^−1^ (ref. ^24^).

In this work, we show that the marriage of a permanent micromagnet and a commercial accelerometer can realize a single-point MEMS gradiometer with a high gradient field resolution (100 pT cm^−1^ in vacuum and 1 nT cm^−1^ in air at resonance) and high spatial resolution (250 µm magnetic sensing element). The calculated noise floor is 110 fT cm^−1^ Hz^−1/2^, well within the range of biomagnetic field sensing^25^. The key developments enabling the sensor discussed in this work are the highly engineered MEMS accelerometer and the permanent micromagnet. The capacitive accelerometer is the classic success story of the MEMS industry, fulfilling a need in the automotive market for sensitive, low-cost detection for airbag sensors^26^. These lucrative applications have driven the development of MEMS accelerometers, reaching resolutions of 110 µg Hz^−1/2^ for the ADXL203 and 20 µg Hz^−1/2^ for the ADXL354 at costs of $25 and $35 per sensor, respectively^27,28^. At the same time, market demand from the hard disk drive industry and others has pushed the development of rare-earth permanent magnets. High anisotropy, small size, high remanence, and a large variety of coatings for automotive, medical, and consumer products have led to diverse commercial availability^29^. At first glance, the benefits of combining these technologies into a sensor are low power consumption, a small size, and low cost.

The greatest advantage, however, is that permanent magnets, when constrained from rotating, are sensitive only to the forces from gradient fields. Therefore, while the Earth’s field is large (50 µT), its spatial gradients are small, and the gradient noise is even smaller at 500 fT cm^−1^ (ref. ^30^), reducing the interference. Permanent magnets of large volume and high magnetization cannot be directly integrated into traditional MEMS fabrication, but techniques such as pick-and-place and flip-chip bonding can be used at a reduced throughput^31^.

## Results

### Gradiometer fabrication from MEMS accelerometer

**Fig. 2:**
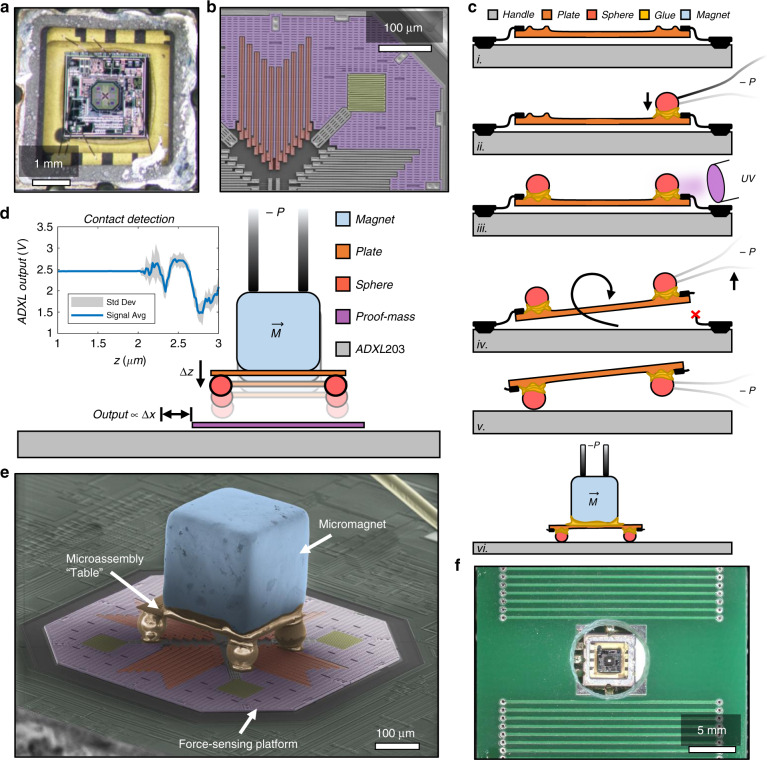
Fabrication of Gradiometer. **a** Optical image of the ADXL203 accelerometer from Analog Devices 28 after hermetic lid removal. The octagonal proof-mass in the center is surrounded by integrated circuitry. **b** Colorized scanning electron microscope (SEM) image of the top-right quadrant of the accelerometer sensor. The proof-mass (purple) is anchored through the springs (yellow), and the position is sensed by interdigitated capacitive fingers (red). **c** Schematic of the subassembly fabrication. The polysilicon plate (i) is designed in-house and fabricated at a foundry (see “Supplementary Materials”). It is mechanically tethered by polysilicon springs (black). A microsphere is manipulated by a micropipette, dipped in UV glue, and placed in a corner of the plate (ii). This is repeated on all corners before the epoxy is cured with UV light (iii). The micropipette is used on one sphere to break the tethers and flip the assembly around to stand like legs under a table (iv–v). A micromagnet is oriented, dipped in UV glue, and then cured in the center of the “table” (vi). **d** Fabrication on a postrelease MEMS. The subassembly is dipped lightly in UV glue and oriented above the ADXL203 proof-mass before being lowered carefully until noise is sensed on the output, indicating contact. It is important that the subassembly is attached without interfering with the natural sensing mechanism of the ADXL203. **e** Colorized SEM image of the fully fabricated gradiometer (not the device used in this work). **f** Gradiometer assembled inside printed circuit board (PCB) antiparallel coils for experimental characterization

Our sensor is a marriage of two matured technologies: the capacitive accelerometer and permanent micromagnets (more in “Methods”). The accelerometer is a sensing platform fit for adaptation to other measurands because it inherently senses the position of a movable polysilicon plate. Figure 2a shows the ADXL203 accelerometer with the hermetic lid removed, revealing the silicon die underneath. The octagon in the center is the proof-mass, a quadrant of which is expanded in the false-colored scanning electron microscope (SEM) image shown in Fig. 2b. The proof-mass (purple) is a polysilicon plate that can be mechanically coupled to a variety of microscale objects, functionalizing the device for other sensing applications^32^.

A custom pick-and-place tool and procedure were developed for the assembly of microscale objects on the proof-mass, as illustrated in Fig. 2c, d (see the “Supplementary Materials” for more information). A fully fabricated sensor is shown in Fig. 2e as an SEM image. A permanent magnet distorts the SEM image; thus, the device shown is for illustrative purposes only, where the magnet is completely demagnetized and the UV glue under the spheres overlaps some portions of the spring (compromising the sensing ability). Similar fabrication techniques have been used to develop an MEMS Casimir force metrology platform^32^, a full hemisphere, tip-tilt micromirror^33^, and other MEMSs at Nokia^34^. Figure 2f shows a sensor fabricated within a custom printed circuit board (PCB) coil for gradient field characterization.

### Electrostatic characterization

**Fig. 3:**
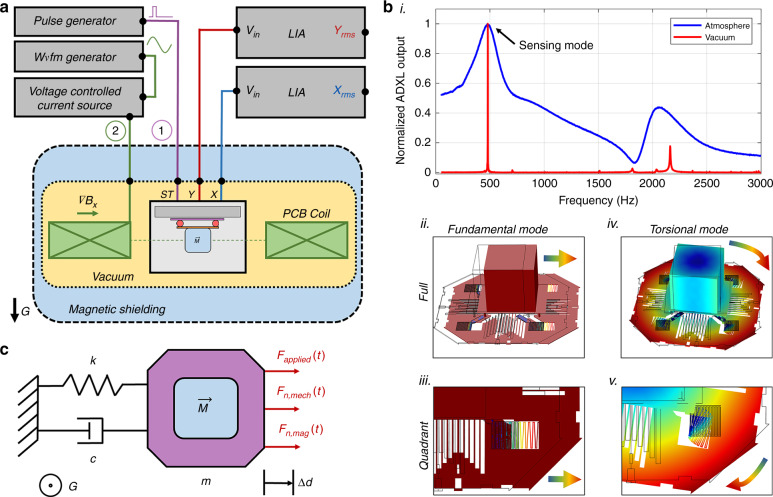
Experimental setup and electrostatic characterization. **a** Gradiometer on a PCB coil assembly is oriented upside-down in a chamber with the option to pull the vacuum (yellow) and apply magnetic shielding (blue). Feedthroughs provide power to the sensor and PCB coil as well as sense the outputs of the functionalized ADXL203. A pulse generator is used in combination with a built-in self-test (ST) functionality to electrostatically characterize the sensor mechanics in air and in vacuum (purple, 1). A waveform generator is used in combination with a precision current source and the PCB coil wired in antiparallel to magnetically characterize the sensor with gradient fields (green, 2). Both X (blue) and Y (red) outputs are filtered by lock-in amplifiers. **b** Characterization using the electrostatic drive described in (a) and a COMSOL Eigenmode simulation. A square-wave frequency sweep (i) reveals two actuation modes and demonstrates an increase in the quality factor in vacuum. Full view of the simulation deflection at the first mode, which depicts a translation (ii), and a quadrant view (iii), which depicts spring deformation. Full view of the simulation deflection at the second mode, which depicts the torsion (iv), and a quadrant view (iv), which depicts spring and plate deformation. In all color maps, red is the largest deformation and blue is the smallest. **c** The simplified free body diagram of the translational mode (top view) resembles a damped harmonic oscillator. The applied force can be driven electrostatically or magnetically. Forces due to mechanical and magnetic noise are also shown (represented as Fn)

The experimental setup is illustrated in Fig. 3a. The sensor can be driven by two mechanisms: electrostatically (purple circuit) and magnetically (green circuit). Further details are provided in the Supplementary Materials. The modified ADXL203 is characterized by electrostatic actuation in Fig. 3b i and a COMSOL simulation in Fig. 3b (ii–v). Figure 3b (i) displays the results from a square-wave frequency sweep (10 Hz to 3 kHz). The duty cycle is 20% in air and 0.02% in vacuum, maximized to achieve the strongest signal without overdriving at resonance. The root mean square gradiometer output is normalized so that the quality and relative peak magnitude can be compared. Two resonant peaks are shown, i.e., one near 500 Hz and another near 2.2 kHz, and the quality factors (sharpness) of the peaks increase greatly from atmosphere to vacuum as the damping is decreased. The 500 Hz peak is expected, as the resonant frequency will decrease from 5.5 kHz when mass is added (see “Methods”). The quality of the 500 Hz peak is 2 in atmosphere and >1000 in vacuum, demonstrating increased sensitivity at resonance when viscous damping is reduced. Figure 3b (ii–v) qualitatively illustrates the two modes using the COMSOL Eigenmodes tool. Materials and geometry are input into the model, resulting in a calculation and visualization of the mode frequency and deformation, respectively. A 3D computer automated design file of the ADXL203 proof-mass is generated from an SEM image, where the thickness is measured to be 4 µm. In the simulation, the proof-mass is rigidly attached to the magnet-table subassembly, constrained by a roller in the XY plane, and anchored at four points in the center. For this input configuration, a translational mode at 600 Hz and a torsional mode at 1.5 kHz are found. Errors in the mode frequency simulation are likely due to inaccuracy in the model geometry and the assumptions of material properties. Figure 3b ii shows a full device view of deformation at the lower frequency mode, and Fig. 3b iii shows the same mode, cropped and oriented so that the spring deformation in a quadrant of the proof-mass can be visualized (red indicates the largest deformation, while blue indicates the smallest). The deformation is translational along the *x*-axis, i.e., the direction of magnetization. This is the type of deformation we would expect from a force imposed by a gradient magnetic field (see “Methods”, Eq. 4). Similarly, Fig. 3b iv–v shows a torsional deformation at the higher frequency mode, where the assembly torques about the center of the x–y plane. This is the deformation we would expect from a uniform magnetic field in the x–y plane but not oriented along the magnet’s dipole axis (see “Methods”, Eq. 7). Since we are imposing a magnetic field with no uniform component and a constant gradient along the *x*-axis, we are primarily interested in the effect seen at the translational mode, and we can disregard the higher torsional mode. Based on this electrostatic characterization, we can simplify our mechanistic understanding of the sensor to a one-dimensional, underdamped harmonic oscillator model (more information given in the Methods section), the free body diagram of which is illustrated in Fig. 3c. The collective mass of the subassembly and the proof-mass are treated as a rigid body with mass *m*. The four springs on the proof-mass are lumped into a single spring constant, *k*, and damping in air or vacuum modulates the constant *c*. An applied force (electrostatic or magnetic) along the *x*-axis results in a displacement along the same axis. Forces from mechanical and magnetic noise are posited to limit the experimental resolution of the device (see “Discussion”).

### Magnetic characterization

**Fig. 4:**
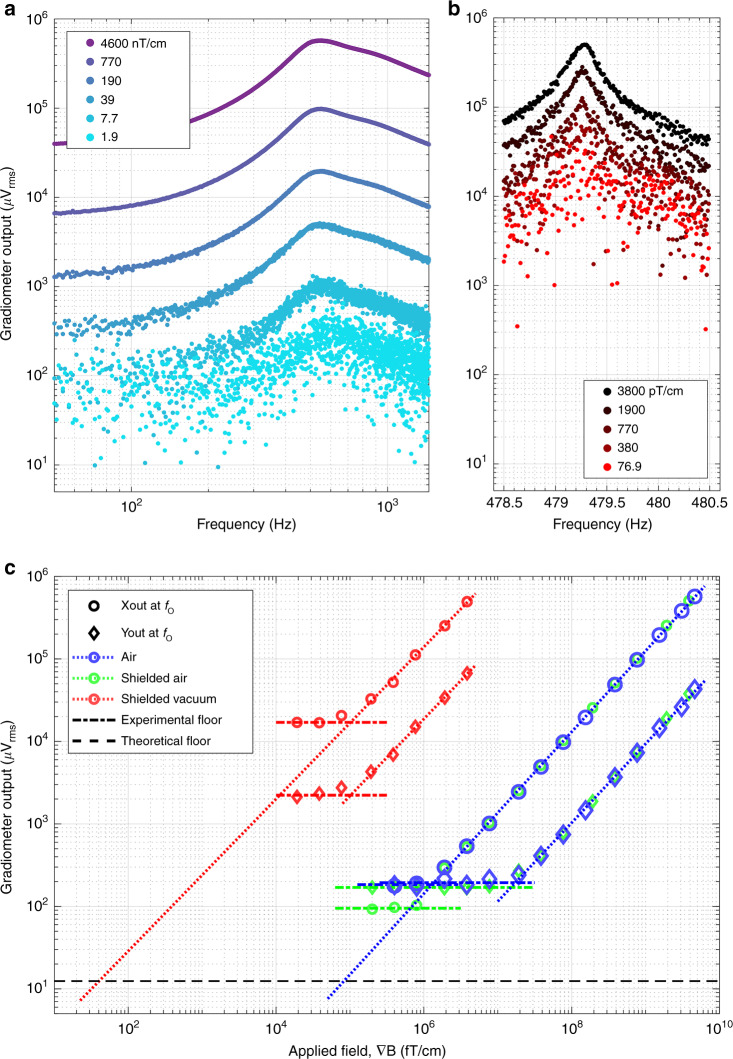
Magnetic sensing performance. **a** Broad sine wave frequency sweep of the gradient field in air (around the translational mode). Sensor output decreases monotonically with the peak-to-peak of the imposed gradient magnetic field. **b** Narrow sine wave frequency sweep of the gradient field in vacuum (around the translational mode on the tip of the high-quality peak). Sensor output decreases monotonically with the peak-to-peak of the gradient magnetic field. **c** Gradiometer output at the peak of the sweeps in **a**, **b** for air, shielded air, and shielded vacuum conditions. Signals along the magnetic direction *x*-axis (circles) are over an order of magnitude higher than those along the *y*-axis (diamonds), indicating good magnet alignment and low cross-axis sensitivity. A linear least squares fit is conducted on data above the experimental floor to determine the sensitivity (slope). All sensitivities are nearly 1 µ Vrms (fT cm^−1^)^−1^. Both axes in air reach a floor at the same sensor output, indicating a limitation of mechanical or electrical noise. Both axes in vacuum reach a floor at the same gradient magnetic field strength, indicating a limitation of the gradient magnetic noise. In air with shielding, the *x*-axis reaches a lower sensor output floor than does the *y*-axis, demonstrating the attenuation of magnetic noise. Based on the linear fits, the gradiometer’s resolution is 100 pT cm^−1^ in vacuum and 1.1 nT cm^−1^ in air. The linear fit can be extrapolated to the noise floor of the device, based on the ADXL203 noise density28 (see “Methods”)

The gradiometer’s performance is dynamically characterized in three conditions: air (case 1), air with magnetic shielding (case 2), and vacuum with magnetic shielding (case 3). In all cases, the frequency of the gradient field and bipolar sine wave are swept as the gradiometer output signal is processed by lock-in amplification. The gradiometer output voltage is proportional to the gradient magnetic field by Eq. 8 (“Methods”). Figure 4a shows the results from case 1, where the frequency is swept from 50 Hz to 1.1 kHz and the field strength is swept from 4.6 µT cm^−1^ to 1.9 nT cm^−1^. Similar to electrostatic characterization, a low frequency peak is again present near 500 Hz, indicative of the translational mode and displacement along the *x*-axis. The largest applied field is 4.6 µT cm^−1^, as higher fields result in a clipped output signal at resonance by the ADXL203 conditioning circuit. The sweeps follow a monotonic pattern, decreasing in signal output as the field magnitude is decreased. At lower field magnitudes, the signal-to-noise ratio visibly diminishes and is eventually overcome by noise. Figure 4b shows the results from case 3, where the applied field is swept in a narrower frequency range on the tip of the high-quality peak (478.5 Hz to 480.5 Hz) and in a field range from 3.8 nT cm^−1^ to 76.9 pT cm^−1^. Again, the largest applied field shown is 3.8 nT cm^−1^, above which output signals are clipped by the ADXL203. The sweeps again follow a monotonic pattern corresponding to the field magnitude. The resonance is approximately 479.2 Hz. Figure 4c displays the gradiometer output at resonance with respect to the applied gradient field, processed from the sweeps conducted in Fig. 4a, b. Here, data from sweeps below the experimental resolution of the sensor are included to characterize the experimental noise floor. Data from case 2 (green) are now included and are largely similar to those in case 1, except with a lower resolution along the sensing axis. Data from case 1 and case 3 correspond in color to Fig. 4 a, b, respectively. The characteristics from each case are also tabulated in Table 1. Circles represent the output from the *x*-axis (along the magnet’s dipole axis), while diamonds are the sensor *y*-axis output. In all cases, the *y*-axis is also sensitive to the applied field but is lower than 14% of the x-output, indicating good magnet alignment and reduced cross-axis sensitivity. The linear dynamic range of the gradiometer output in fT cm^−1^ is 3.3 decades in case 1 and 1.4 decades in case 3. The dotted lines show a linear least square fit of data above the experimental noise floor, where the sensitivity, *γ*_mag_, is consistently linear and near 1 µVrms (fT cm^−1^)^−1^ in all cases. The black dashed line represents the noise floor, scaled from the ADXL203 noise density with optimal lock-in filtering (“Methods”). The dotted lines are extrapolated to the noise floor to show the calculated noise floor in air and vacuum. The dash-dot lines are a zero-order, least square fit of data below the experimental resolution, representing an experimental floor in each case. The intersection of the dotted line and dash-dot line is the experimental resolution of the sensor (Table 1). It is most noteworthy that the X- and Y-outputs have the same experimental floor in case 1 and reach an experimental floor under the same magnetic field in case 3. In case 2, the X-output extends lower than both the Y-output and case 1 data. These relationships are indications of resolution-limiting noise, which is elaborated further in the “Discussion” section.

**Table 1:** Sensor performance metrics based on condition

Case	Condition	Experimental resolution (pT/cm)	Theoretical resolution (pT/cm)	*γ*_mag_, linear sensitivity X (µV/fT/cm)	Cross-axis sensitivity (%)	Range (10^n^ fT/cm)	Experimental floor *X* (µVrms)	Experimental floor *Y* (µVrms)
1	Air	1,050	60	0.98	6.6	3.3	180	190
2	Shielded Air	700	60	0.99	7.5	3.6	95	170
3	Shielded Vacuum	100	0.03	0.92	13.7	1.4	17,000	2200

Tabulated values are extracted from the plot in Fig. 4c

**Fig. 5:**
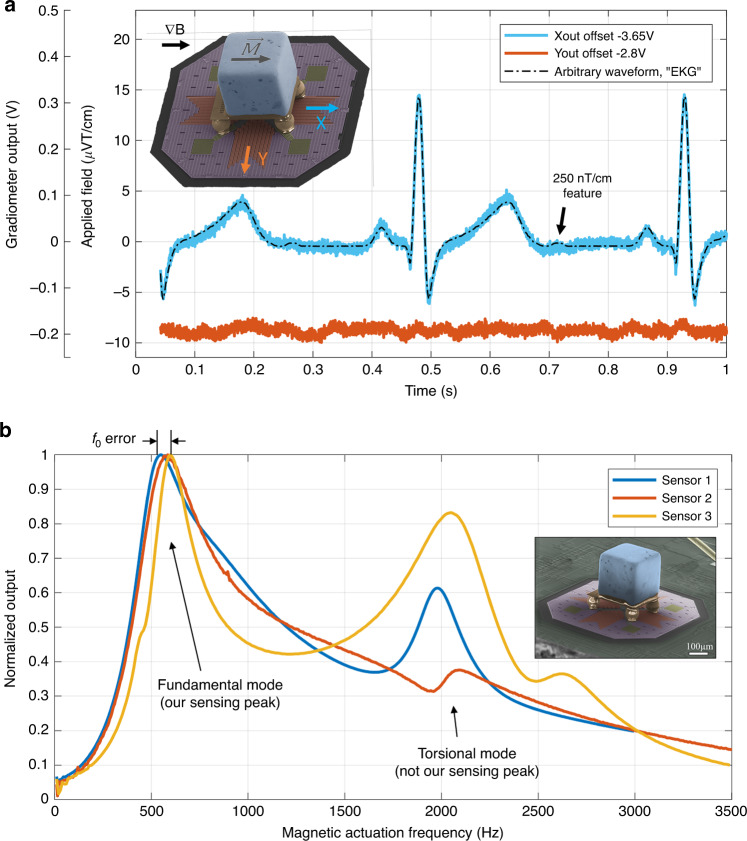
Waveform demonstration and fabrication repeatability in air. **a** Raw output of magnetic sensor in air in response to an arbitrary waveform resembling an electrocardiogram (EKG) at 2.2 Hz and 20 µT cm^−1^ peak-to-peak, imposed along the *x*-axis by a PCB coil. The sensor output is displayed in terms of both the voltage and gradient magnetic field. Both X and Y outputs are offset for ease of visualization. The x output tracks the imposed field very well, while the Y output does not resolve any of the features in the magnetic signal. The inset (top left) illustrates the magnet alignment with respect to the sensitive *x*-axis and the insensitive *y*-axis. **b** Comparison of three different sensors based on magnetic frequency characterization in air. All sensors present both the fundamental and torsional modes, with some variability in the resonant frequencies and quality factors

The raw, unprocessed performance of the sensor in air is illustrated in Fig. 5a, combining some of the performance metrics displayed in Table 1 (case 1). It is also important to highlight that the data in this plot are not taken at resonance, where the signal-to-noise ratio is far more favorable. Rather, it corresponds to a lower frequency regime, where many common biomagnetic signals exist. An arbitrary waveform (black, dashed) resembling a magneto-cardiogram is imposed as a gradient field signal at the low frequency of 2.2 Hz. Within a period, the signal is composed of higher frequencies, most of which are below 60 Hz. The blue data are the raw output from the magnetic axis and are shown to track the gradient signal very well with no distortion. The red data below the waveform show the output of the Y-axis, showing only mechanical noise and no features from the arbitrary waveform. The inset in the top left is an SEM of the gradiometer showing the *x*- and *y*-axis directions, where the *x*-axis is the magnetized direction. Both axes are offset on the plot for ease of visualization. The biomagnetic signatures are typically in the hundreds of pT cm^−1^, and the signal shown here is a 20 µT cm^−1^ peak-to-peak one (the smallest feature is a 250 nT cm^−1^ peak indicated at 0.7 s). While this is several orders of magnitude away, the Discussion expands on why this is a promising platform for these measurements in the future.

The dynamic response of three different devices with the same design and fabrication method is presented in Fig. 5b (in air; magnetically actuated). It can be seen that the fundamental and torsional modes are present at nearly the same frequencies for each sensor. The error in the fundamental mode between 550 and 600 Hz translates to a difference in mass of 20 µg. The quality factors of the fundamental modes range from 1.2 to 1.5, suggesting good agreement. The magnitude of the torsional mode peak cannot be directly interpreted since the sensor is primarily designed to sort motion into X and Y displacements. However, the magnitude and quality of this peak may be a good gauge of the overall symmetry and alignment in a sensor. Sensor 1 has the cleanest output here and is the device characterized in the rest of this work.

## Discussion

### Experimental and design limitations

It is noteworthy that several asymmetries result from this manual fabrication process that could limit the resolution of the sensor. Some of these include a displaced center of mass (anisotropy of the cube magnet geometry, centering of a magnet on a table, table on a proof-mass), variable sphere size and area of contact, magnet orientation, magnetization direction, and contamination of the sensor by opening the sealed package. With the added weight of the micromagnet, these asymmetries may manifest themselves by pulling the proof-mass out of plane with respect to the capacitive fingers, which are designed to detect in-plane displacement only. Any rotational assembly error or uniform field (such as the Earth’s field) may create an offset torque of the proof-mass, creating greater asymmetry in the springs and mechanical motion. For this sensor to reach less stationary applications, several improvements to its robustness are necessary. Large spikes in the magnetic field could damage the device by providing a large torque (uniform field spike) or force (gradient field spike), either breaking the magnet from the proof-mass or breaking the proof-mass from the device. Similarly, large mechanical shocks, such as dropping the device, could result in similar damage. The micromagnet used in this work was very small, but its weight has the potential to affect the results in other prototypes. As such, the measurements were conducted with the sensor inverted; thus, the magnet was underneath and centered. This configuration was used to prevent the negative effects that the weight of the magnet could have on bringing the proof-mass into contact with the handle or to prevent an offset of the magnet to one side or the other. If a specialized MEMS proof-mass was designed, the springs could be stiffened in the out-of-plane direction to reduce the deflection as well as difficulty during fabrication.

The fabrication method presented here is low-throughput and useful mainly for prototypical design and proof-of-concept work. However, higher-throughput fabrication techniques exist to accomplish similar tasks, such as pick-and-place or flip-chip bonding^31,35^. One of the greatest challenges of this work was handling a permanent micromagnet and attaching it with other microstructures to a delicate, postrelease MEMS device. It should be noted that the UV epoxy used is relatively HF resistant; thus, higher-throughput techniques could be used to fabricate the sensor prerelease. Furthermore, the presented design will add challenges to the construction of multiaxis gradiometers, as magnets on neighboring devices can cause interference. More work is needed to determine the minimum sensor spacing and interference. Other MEMS designs, such as Lorentz force sensors, are more amenable to 3-axis measurement with a single sensor, such as a device that uses multiloop recirculation within the resonant structure^13^.

### Exclusive sensitivity to gradient magnetic fields

The mechanical modes of our gradiometer are characterized by electrostatic actuation in Fig. 3b (i) and by a mode simulation in Fig. 3b (ii–v). We argue that the fabricated sensor is sensitive only to gradient magnetic fields, which impose a force along the dipole axis of a magnet and result in translational deformation (Methods). Sensitivity to uniform fields will result in a torque of the magnet. This is supported by the simulated mode deformation in Fig. 3b (ii), where we show that the fundamental mode is a translational deformation along the dipole axis of the magnet. Thus, any information from this mode will come only from gradient magnetic fields. We also explain the prediction of the 500 Hz fundamental mode from the simplified free body diagram in Fig. 3c and Eq. 2 (“Methods”). Furthermore, we show that there is a separate, higher mode of torsional deformation (Fig. 3 (iv)) about the center of the magnet that corresponds to a uniform field. This shows that information at the fundamental mode is not directly affected by uniform fields. The magnetic characterization of the device, then, is centered around the fundamental mode.

### Resolution-limiting noise

The magnetic characterization in Fig. 3c and Table 1 reveal differing resolutions in each case and on each output axis of the fabricated sensor. Analyzed together, the experimental noise floors are suggestive of the type of noise that limits the resolution. The limiting noise is considered for each case below.

Case 1

In case 1 (air without shielding), the experimental floors of both the X- and Y-outputs coincide at the same sensor voltage, indicating that both axes are subject to common noise. Since the noise floor is independent of the magnet orientation, it cannot be due to the gradient magnetic field noise, which would predominantly actuate the magnet’s central axis. Any asymmetries from fabrication discussed earlier could result in common electrical noise on the output from out-of-plane capacitive fingers. The directionality detection of the ADXL203 is designed to modulate each axis differently, and out-of-plane deformation can enhance the cross-talk of these signals. Furthermore, the device is unshielded in case 1, and an offset torque from the Earth’s uniform field can add to the asymmetry. The mechanical vibrations of the experimental setup typically follow 1/f but contribute to both axes of the sensor. Thermomechanical noise, resulting from the Brownian motion of air molecules, will also be common to both axes. This is a white noise that will present random fluctuation of the forces as air molecules interact with surfaces such as the side of the magnet. The magnet presents a large surface area and is therefore a large source of this noise in air. Since the magnet is well oriented with a face along the *x*- and *y*-axes, the noise will be similarly symmetric. Last, a well-known effect of MEMS resonators is squeeze film damping, an effect derived from the many small gaps (<5 µm) between capacitive fingers. Here, the same thermomechanical (viscous) damping is presented on the fingers. Second, elastic damping of the thin air gap results in stiffening of the structures and nonlinear effects^36^.

Case 2

When shielded in air (case 2), the X-axis reaches a lower resolution (from 1.1 nT cm^−1^ in case 1 to 700 pT cm^−1^ in case 2), while the Y-axis remains the same. The effect of the shielding may correct an offset torque from the Earth’s field that could be added to asymmetry, as discussed in case 1. Additionally, the magnetic shielding will present an asymmetric magnetic damping of both the actuation signal and the micromagnet. The lower noise floor of the *x*-axis may be attributed to this. It is unlikely that uniform geomagnetic noise plays a role here, as geomagnetic noise is far lower at 100 pT (ref. ^1^).

Case 3

The results from the shielded vacuum condition (case 3) reveal a common experimental floor of both sensor axes at the same applied field rather than the same sensor output voltage. This indicates that the limiting noise source is different than that in case 1 and case 2. The *y*-axis motion can still be detected at displacements much lower than the x-axis, suggesting that thermomechanical damping is not the limiting noise, as it would act symmetrically. Therefore, the limiting noise must be gradient magnetic noise. Possible sources are either geomagnetic or the gradient coil driving system. Geomagnetic gradient noise has been reported to be much lower at 500 fT cm^−1^ (ref. ^30^). However, measurements were conducted during daytime hours in a major city. Therefore, gradient noise from the environment may be larger than that presented in this report but is likely not the limiting noise. The voltage-controlled current source (CS580) is specified to have superb output noise characteristics (60 fA Hz^−1/2^ in the configuration used). However, the instrument is some distance away from the PCB coils, the wire is carried next to all other sensor leads with relatively small drive currents (<100 nA drive, 11 mA power), and connections are made at vacuum chamber feedthroughs. This may make the drive signal vulnerable to pick-up or cross-talk, which is amplified and superimposed onto the magnetic driving force, thus limiting the sensor resolution.

### Present and future applications

The sensor is characterized in three conditions (Table 1) to demonstrate its performance in the context of various applications. Most applications exist in ambient conditions and do not require the enhanced performance that a vacuum and shielding provide. For example, dipole sources, such as planetary magnetic fields or ferromagnetic objects, have gradient signatures that are difficult to measure with a uniform field sensor alone. Among these applications, our gradiometer offers the key advantage of directly sensing the gradient field rather than the difference between two uniform field sensors. Moreover, it is capable of doing all this with a small size, with low power, at a low cost, and at room temperature. Finally, the realization of the thermomechanical noise floor of the ADXL203 will offer the unique ability to sense biomagnetic fields in ambient conditions, an idea that is attractive for wearable sensing (such as the signals illustrated in Fig. 5a).

For the most sensitive applications, a vacuum or shielding can be applied. The vacuum can be pulled on the resonant sensor, as shown in Figs. 3b (i) and 4b, to increase the quality and resolution of the fundamental mode. At scale, vacuum packaging is a solved problem for the MEMS^35,37^, which allows for enhanced resolution at a small scale. Resonant mode operation is typically a design tradeoff, limiting a sensor to a specific, narrow band of frequencies around resonance. However, a resonant sensing mechanism does not necessarily impede a resonant sensor from identifying features at other frequencies of interest. MEMS actuators with flux guides have been designed to modulate an arbitrary signal so that it can be measured at the resonant frequency of a sensor^6^. Others have leveraged a nonlinear spring stiffness during cyclic resonant motion, ultimately reporting a shift in resonant frequency instead of the oscillation amplitude^37^. Furthermore, others have made progress regarding the integration of magnetic materials with MEMS in a process called M&NEMS^38^. Shielding becomes useful when the target of measurement can also fit inside the shield. For this, shielded rooms with 60 dB attenuation are common and often used for biomagnetic measurements. We report the effect of only 20 dB magnetic attenuation, which demonstrates the potential for enhanced resolution with greater shielding. Finally, the combination of an improved vacuum, shielding, and environment can enable reaching a thermomechanical noise floor of the ADXL203 (13 fT cm^−1^) directly on par with that of SQUIDs and optically pumped, atomic magnetometers (Fig. 1a). This sensor is disruptive in cost, size, and gradient sensing mechanism, making the approach applicable to the most sensitive applications, such as biomagnetic fields.

## Conclusions

The purpose of this work was threefold: (1) to build a magnetic sensor that is sensitive only to gradient magnetic fields, (2) to demonstrate repeatability and a wide field strength and frequency space, and (3) to achieve 1 and 2 in a small, low-cost, and commercially available platform. We have characterized the performance of a new MEMS magnetic gradiometer with a resolution of 100 pT cm^−1^ in a shielded vacuum and a range spanning over 3 decades under ambient conditions (1.1 nT cm^−1^ to 4.6 µT cm^−1^). Compared to existing MEMS magnetic sensor designs, our resolution presents a 30-fold improvement compared with that of MEMS magnetometer technology^17^ and a 1000-fold improvement compared with that of MEMS gradiometer technology^24^. The sensor has a small spatial resolution based on a 0.25 mm magnetic sensing element and a total sensor-plus-package footprint under 10 mm^2^. More sensitive accelerometers, such as the ADXL354, will theoretically be able to improve this sensitivity by a factor of ten^28^. We have achieved all of this on a small, versatile platform, which is easily integrable into consumer technology integration. This new technology has the potential to revolutionize magnetic sensing while also offering many advantages to other fields, such as navigation, communication, and biomagnetic field mapping.

## Materials and methods

### MEMS accelerometer

Figure 2b shows an SEM image of a quadrant of the ADXL203 from Analog Devices. The spring (yellow) is nearly symmetric on both the *x*- and *y*-axes. Displacement is sensed via capacitive fingers (red) in a differential configuration. We chose the ADXL203 for its intrinsically low noise density (110 µG Hz^−1/2^), linearized sensitivity (1 V G^−1^), wide range (up to 10^4^ G with optimized filtering), and accessible proof-mass^10^. From experimental observations, the two-axis accelerometer was found to have a resonant frequency of 5.5 kHz, a spring constant of 1 N m^−1^, a Q in air of 10, and a Q in vacuum of 10,000. The maximum sensing range in one direction was 25 nm, giving a sensitivity of 10 nm V^−1^. This means that the device has a noise density of 1 pm Hz^−1/2^, or 1 pN Hz^−1/2^.

### Permanent micromagnets

Permanent micromagnets are a sintered mix of rare-earth element powders and are typically coated for protection or passivation^29^. The micromagnets in this work are cubes and magnetized to N52 grade (SM Magnetics Co.). The smallest commercially available cube magnet of 250 µm side length was chosen to minimize gravitational forces and optimize the spatial resolution. The powders consist of neodymium, iron, and boron. The standard coating of nickel, copper, and nickel was used to avoid degradation.

### Measurement theory

#### Governing mechanics

A simplified, one-dimensional mechanical model is shown in Fig. 3c. The gradiometer behaves as an underdamped harmonic oscillator (Eq. 1), with settling times of 20 ms in air and 3 s in vacuum (within 2% of the final value).

1 $$m\ddot d + c\dot d + kd = F_{{\mathrm{applied}}}\left( t \right) + F_{n,{\mathrm{mech}}}\left( t \right) + F_{n,{\mathrm{mag}}}(t)$$

The system may be thought of as one-dimensional because the ADXL203 has a low cross-axis sensitivity (1.5%) between X and Y^27^ and because out-of-plane forces are minimized by centering the magnet on the proof-mass using the table subassembly. The magnet, table, and proof-mass are collectively considered to be one rigid body. Together, their mass is found to be 160 µg from the relationship between resonant frequency and mass shown in Eq. 2. The resonant frequency is found from an electrostatic frequency sweep, as shown in Fig. 3b (i). This explains the effect of a decreased resonant frequency when mass is added. The spring constant, k, is estimated to be near 1 N m^−1^. First, the volume of the proof-mass is estimated from SEM images of the structural dimensions. Assuming a polysilicon material, the density can be used to estimate the mass. This mass and the resonant frequency without added mass (5.5 kHz) are used in Eq. 3 to find k.

2 $$m = \frac{k}{{(2\pi f_0)^2}}$$

The constant c in Eq. 1 represents damping. The vacuum decreases damping and is shown to increase the quality factor in Fig. 3b i. At resonance, this increases the amplitude of oscillation, *d*_*f0*_. For a constant force at resonance, *F*_*f0*_, the amplitude increases proportionally to the quality factor, *Q*, as shown in Eq. 3 (ref. ^26^).

3 $$d_{f0} = Q\frac{{F_{f0}}}{m}$$

#### Forcing and magnetics

The applied force, *F*_applied_, is proportional to the gradient field^26^ generated by the PCB coil (Eq. 4) as described in the previous section. The micromagnet has a moment, $\vec M$, of 15 µJ T^−1^, calculated from experimental data and confirmed by a simulation. The permanent magnet is approximated as a dipole in Eq. 5, and the data are fit by a cubic function^26^. The experimental magnetic field, B, is gathered from a Hall sensor along the central axis of the magnet, r. The constants, including the magnetic permeability of free space, M_0_, are condensed to *α*, and the moment of the magnet, M, is extracted. This is confirmed by a simulation using the finite element methods magnetic (FEMM) software and a 250 µm cylindrical magnet of N52 grade. Again, the magnetic field, B, is collected at various distances along the central axis, *r*, and fit by a cubic function to extract the moment, M.

4 $$F_{{\mathrm{applied}}}\left( t \right) = M \cdot \nabla B(t)$$

5 $$B\left( r \right) = \frac{{2M_0M}}{{4\pi }}\frac{1}{{r^3}} = \frac{{\alpha M}}{{r^3}},$$

The main sources of noise are mechanical, *F*_n,mech_, and gradient magnetic, *F*_n,mag_, but these are insignificant for large fields and thus are analyzed in the Discussion section as resolution-limiting terms. The effect of gravity is ignored since the gradiometer is held upside down, and any gravitational forces are outside of the sensing plane. This simplified model is relevant only to the fundamental mode in Fig. 3b (ii), as the mode described in Fig. 3b (iv) is deforming in two dimensions. This torsional deformation can be actuated by a uniform magnetic field, similar to a compass. The relationship between a uniform field, B, and the magnet with moment M is a torque, T, as shown in Eq. 6 (ref. ^39^). The ADXL203 is designed to sense motion in either the X or Y directions, however. Therefore, in this case, the sensor will not have a meaningful output.

6 $$T = M \times B$$

#### Sensor transduction

The ADXL203 directly measures a differential capacitance, which is inversely proportional to a displacement of the proof-mass (in either the X or Y directions, see Fig. 2b). The sensor is linear in the measurement range; thus, its signal output, *S*, is related to the displacement, *d*, by a proportionality constant, *γ*, in Eq. 7. In the linear regime of the springs, Hooke’s law relates displacement to a force. Equation 4 shows that ∇*B* is proportional to a force, *F*_applied_. Once functionalized with a micromagnet, the sensor output, now *S*_mag_, is then proportional to the applied ∇*B* by the sensitivity, *γ*_mag_ (Eq. 8). The sensitivity of the gradiometer is 1 µV (fT cm^−1^)^−1^ from the experimental measurement in Fig. 4c and is tabulated in Table 1.

7 $$S = \gamma d$$

8 $$S_{{\mathrm{mag}}} = \gamma _{{\mathrm{mag}}}\nabla B$$

#### Noise floor calculation

Using the understanding of the mechanics and the effect of magnetic fields on the sensor output, the noise floor can be calculated. The calculation is based on the thermomechanical noise floor of the sensor. The ADXL data sheet reports a noise density, *ρ*, of 110 µV Hz^−1/2^ (ref. ^27^). This accelerometer noise density can be scaled by the magnetic sensitivity, *γ*_mag_, shown in Eq. 9 to find the magnetic noise density, *ρ*_mag_. This yields a magnetic noise density of 110 fT cm^−1^ Hz^−1/2^.

9 $$\rho _{{\mathrm{mag}}} = \gamma _{{\mathrm{mag}}}\rho$$

The equivalent noise bandwidth (ENBW) when using a 24 dB oct^−1^ roll-off and 300 ms time constant is equal to 0.26 Hz. The minimum ENBW for the most sensitive measurements is 0.008 Hz. The resolution at a given frequency is calculated from the noise density, $\rho$, by Eq. 10 (ref. ^40^).

10 $${\mathrm{Resolution}} = \rho \times \sqrt {{\mathrm{ENBW}} \times 1.6}$$

## Supplementary information

Supplemental Material

Supplementary Table 1
