# Supplementary material for: 100 pT/cm single-point MEMS magnetic gradiometer from a commercial accelerometer
Source: Microsyst Nanoeng. 2020 Aug 10;6:71. doi: 10.1038/s41378-020-0173-z (PMC8433323; doi:10.1038/s41378-020-0173-z)
Supplement: Supplementary file 1 — Supplemental Material [file 41378_2020_173_MOESM1_ESM.docx]

# State-of-the-Art Lorentz Force MEMS Magnetometers

In the course of this work, it was helpful to review recent advancements of other MEMS magnetometers, such as those that utilize the Lorentz force for detection^12-16^. Similar to our work, these sensors are beneficial for their small size, low power, and (depending on the application) design bandwidth. Size is particularly beneficial because field magnitudes tend to increase nearly a cubic function with distance in most cases, and small sensors are able to be closer to the measurand. In **Supplementary Table 1** below, we show a comparison of recent work to our single-point MEMS gradiometer. It is noteworthy that our sensor is a gradiometer and so the uniform field techniques listed would need to be converted to a gradient configuration as described in **Fig. 1 a** and **b** in order for complete comparison.

**Supplementary Table 1: State-of-the-Art MEMS Lorentz Force Magnetometers.** Work from recent MEMS uniform field sensors is compared in more detail to our MEMS single-point gradiometer.

## Magnetometer Fabrication

The magnetometer is fabricated in two stages. First, a subassembly is made that resembles a magnet on a table (**Fig. 2c**). Second, the subassembly is attached to the post-release MEMS (**Fig. 2d**). The separate subassembly comprises spheres, a polysilicon plate, and a micromagnet. A custom pick-and-place system is used to manipulate and assemble these micro-objects. Vacuum (typically -2psi) is pulled on a glass pipette with the orifice in contact with the object of interest. A micromanipulator on a probe station (Cascade Microtech EPS150FA) and a 3D printed part are used to direct the motion of the pipette in 3 dimensions. A straight pipette (WPI, 30 µm aperture) is used to manipulate microspheres and a custom, 45° angled pipette (Clunbury Scientific, 135 µm aperture) is used with the cube magnet. Borosilicate glass microspheres (Cospheric) of about 65 µm are used as they are sufficiently larger than the pipette, but still small enough to minimize contact with the MEMS proof-mass later. The plate is designed in-house and manufactured by the MEMSCAP foundry process, PolyMUMPs (**Fig. 2c i**). Mechanical tethers of polysilicon are attached to both the plate and the silicon handle so that the plate is suspended when a sacrificial layer of oxide is removed underneath by etching with hydrofluoric acid.

Spheres are assembled on the plate to form a "table," which the magnet sits on. The spheres minimize contact surface area between the micro-objects and the sensitive proof-mass, allowing for repeatable, robust assembly. To assemble a sphere on the plate, vacuum is pulled while in contact with a sphere, which is then wetted on the bottom side with UV glue (Norland Adhesives, NOA 81). The sphere is then positioned above and lowered onto a corner of a plate (**Fig 2c ii**), where it is partially cured by UV light at the manufacturer recommended wavelength of 365nm (Dymax BlueWave) for 15 seconds (**Fig 2c iii**). This is repeated until all four corners of the plate contain spheres. Next, the vacuum pipette is put in contact with one sphere to form a "ball in socket" joint. The pipette is lifted to break the mechanical tethers holding the plate (**Fig 2c iv**). The newly assembled “table” is turned to sit on its legs (**Fig 2c v**). Next, a micromagnet is oriented on a vertical glass slide by an external magnet some distance behind the slide. The larger, angled pipette is brought into contact with the top face of the magnet, which is not one of the poles. The external magnet is removed, leaving magnet on the end of the pipette, held by vacuum. The bottom of the magnet is then dipped in UV glue, aligned on the standing table, and radiated with UV light.

Now that the subassembly is a single rigid structure, the large, angled pipette can be used to attach it to the post-release MEMS. The sensor lid is removed and power is supplied so that the noise on the X and Y outputs can be monitored. The noise is very low normally, but spikes when contact is made by the pick-and-place system, presumably from vibrations in the pipette (**Fig. 2d** inset). The subassembly structure is lifted up and aligned over the center of the proof-mass under a brightfield microscope. The spheres are in predefined locations on the plate so that they make contact with a strip of the proof-mass between the capacitive fingers and the spring. The spheres on the bottom of the plate are dipped very lightly in UV glue, before the structure is carefully lowered toward the proof-mass (**Fig. 2d**). A spike in the accelerometer output signal is used to detect contact between the subassembly and the proof-mass (**Fig. 2d** inset). The spheres minimize contact with the proof-mass so that epoxy does not wick through the release holes patterned on the proof-mass, in which case the device would be rendered insensitive. The structure is then radiated with UV light for 15 seconds before cutting vacuum to the pipette and lifting off. The entire structure is then baked upside-down (to avoid unintended gluing) at 60C overnight to form a full cure (below manufacturer-recommended maximum operating temperature of magnet).

## Experimental Setup and Measurement

The experimental setup comprises a custom PCB coil, custom vacuum chamber, MuMetal shield (Magnetic Shields Corp.), and an instrument drive system (**Fig. 3a**). The ADXL 203 is surface-mounted on a custom printed circuit board (PCB), on which a PCB coil for magnetic characterization is also attached (**Fig. 2f**). The PCB coil consists of two layers separated by the 1.6 mm thick PCB. The top copper traces can be seen in the image and the bottom traces are only different where the end of each line connects it to the next winding in sequence. The 0.1 mm vias, spaced 1 mm apart, connect the two layers to form a 10 turn coil on either side of the sensor. The PCB coil is rigidly connected to the PCB board to reduce mechanical noise in the output. The coil pair central axis is aligned with the micromagnet's dipole axis (along the accelerometer X-axis) for magnetic drive. Force on the magnet is proportional to the gradient of the magnetic field. By wiring the PCB coil in antiparallel fashion, the resulting magnetic field has a constant slope relative to position across the sensor, and thus a gradient magnetic field. The uniform field, then, is zero at the center of the coils, where the micromagnet is positioned. The sensor-coil assembly is fit into a dual inline pin (DIP) socket within a vacuum chamber built using standard parts (Kurt J. Lesker). For experimental results, the chamber is either at atmospheric pressure or in vacuum (1 mTorr). The sensor is positioned upside down to keep the proof-mass free from contacting the substrate underneath in the event of off-axis fields. The chamber is held between vibration isolating pads on a two-axis vertical stage, allowing for the chamber to be moved in and out of a MuMetal shield, which attenuates 20 dB of imposing fields (Magnetic Shields Corp). This entire assembly is built on a passive hydraulic vibration isolation table.

There are two driving schemes of the magnetic sensor shown in **Fig. 3a** drive schematic: Electrostatic actuation and Magnetic actuation. Electrostatic actuation (circuit 1, purple) leverages the capacitively driven self-test functionality of the accelerometer. Originally designed to test whether the accelerometer is in normal working condition, this function may also be used to drive the sensor to arbitrary positions in one quadrant of the actuation range using pulse width modulation (PWM). In this work, self-test is used to non-magnetically actuate the magnetometer over a range of frequencies, characterizing the mechanics of the device after micro-objects are attached. The self-test pin is driven by a precision pulse generator (SRS DG645), as short duration pulses (<200 ns) are required in vacuum to avoid over-driving the MEMS at resonance. The pulse is 0 to 3 V and duty cycle is 20% in air and 0.02% in vacuum. Magnetic actuation (circuit 2, green) is achieved by driving low noise, small (>100 fA) currents through the PCB coil using a voltage-controlled current source (SRS CS580) and waveform generator (Agilent 33210A). The PCB coil's gradient field is linear with drive current (760 µA (µT cm^-1^)^-1^) and unchanging (< 3%) with frequency in the actuation range (DC to 1 kHz). In both drive schemes, the sensor output is only filtered using a lock-in amplifier (SR830). The reference signal is a 50% duty cycle square wave from either the pulse generator or the waveform generator. The equivalent noise bandwidth (ENBW) when using a 24 dB oct^-1^ roll-off and 300 ms time constant is equal to 0.26 Hz. The minimum ENBW for the most sensitive measurements is 0.008 Hz. Resolution at a given frequency is calculated from noise density, $\rho$, by **Eq 10** (ref. 40), and is elaborated on further in the Noise Floor Calculation section of Methods.

$Resolution=\rho*\sqrt{ENBW*1.6}$ (1)
