# Supplementary material for: 100 pT/cm single-point MEMS magnetic gradiometer from a commercial accelerometer
Source: Microsyst Nanoeng. 2020 Aug 10;6:71. doi: 10.1038/s41378-020-0173-z (PMC8433323; doi:10.1038/s41378-020-0173-z)
Supplement: Supplementary file 2 — Supplementary Table 1 [file 41378_2020_173_MOESM2_ESM.pdf]

| Author | Year | Mechanism | Magnetic element footprint (mm^2) | ASIC? | Power (mW) | Sensing Unit (S.U.) | Experimental Resolution (S.U.)/rtHz | Sensitivity (mV/S.U.) | fo (kHz) |
|---|---|---|---|---|---|---|---|---|---|
| Herrera-May | 2015 | Lorentz and piezoresistive | 1.42E-01 | No | 12 | nT | - | 2.30E-07 | 100 |
| Minotti | 2015 | Lorentz off-resonance | 1.28E+00 | Yes | 0.775 | nT | 400 | 5.10E-04 | 20 |
| Kumar | 2016 | Lorentz and thermal-piezoresistive | 2.00E+00 | No | - | nT | 3.5 | 2.1 | 400 |
| Lara-Castro | 2017 | Lorentz with custom conditioning | 4.20E-01 | No | - | nT | - | 3.20E-07 | 14.3 |
| Marra | 2018 | Lorentz 3-axis, recirculation | 5.30E-01 | No | 0.5 | nT | 100 | 6.70E-04 | 50 |
| This work | 2019 | $F = m \cdot \nabla B$ on commercial platform | 6.25E-02 | Yes | 3.5 | nT/cm | 0.155 | 1.00E+03 | 0.5 |

| | | Air | | | Vac | | | | | | |
|---|---|---|---|---|---|---|---|---|---|---|---|
| Author | Year | Resolution (S.U.) | 3dB BW (Hz) | Q | Resolution (S.U.) | 3dB BW (Hz) | Q | P (mTorr) | Resonance Mode | Filter BW (Hz) | Noise Floor (S.U.) |
| Herrera-May | 2015 | 2500 | 250 | 420 | - | - | - | - | Seesaw | - | - |
| Minotti | 2015 | - | - | - | 565 | 20 | 460 | 525 | In-plane expansion | 150 | - |
| Kumar | 2016 | 3.5 | 0.34 | 1E+06 | - | - | - | - | In-plane expansion | - | 0.0028 |
| Lara-Castro | 2017 | 35 | - | - | - | - | - | - | Seesaw | - | - |
| Marra | 2018 | - | 50 | 3200 | - | - | - | "low" | Expansion/seesaw | Lock-in | - |
| This work | 2019 | 1 | 200 | 10 | 0.1 | 0.25 | 600 | 1 | In-plane translation | 0.26 | 0.00003 |
